# Effects of carotid endarterectomy and carotid artery stenting on high-risk carotid stenosis patients

**DOI:** 10.12669/pjms.296.3971

**Published:** 2013

**Authors:** Peifu Wang, Chunyang Liang, Jichen Du, Jilai Li

**Affiliations:** 1 Peifu Wang, Department of Neurology, Aerospace Center Hospital, Peking University Aerospace Clinical College, Beijing 100049, P. R. China.; 2 Chunyang Liang, Bayi Brain Hospital Affiliated to General Hospital of Beijing Military of PLA, Beijing 100700, P. R. China.; 3 Jichen Du, Department of Neurology, Aerospace Center Hospital, Peking University Aerospace Clinical College, Beijing 100049, P. R. China.; 4 Jilai Li, Department of Neurology, Aerospace Center Hospital, Peking University Aerospace Clinical College, Beijing 100049, P. R. China.

**Keywords:** High-risk carotid stenosis, Carotid endarterectomy, Carotid stenting, Security, Treatment efficacy

## Abstract

***Objective:*** To analyze the clinical effects and safety of carotid endarterectomy (CEA) and carotid artery stenting (CAS) in the treatment of high-risk carotid stenosis patients.

***Methods:*** Total 63 patients who underwent CEA or CAS in our hospitals from January 2007 to December 2012 were selected in this study, and were randomly divided into an observation group and a control group. The patients in the observation group were subjected to CAS and those in the control group were subjected to CEA to compare the primary and secondary endpoints of the treatment.

***Results: ***The arrival rates of the primary and secondary endpoints were 7.14% and 10.71% respectively in the observation group, while those were 11.43% and 11.43% respectively in the control group. There were no significant differences in the arrival rates of primary and secondary endpoints between the two groups (P>0.05).

***Conclusion: ***The efficacies and safety of CAS and CEA are similar in treating high-risk carotid stenosis patients.

## INTRODUCTION

Out of all the factors giving rise to stroke, about 20% of intracranial and extracranial lesions are induced by atherosclerosis which should thus be effectively treated.^1^^,^^2^ Compared with drug therapy, carotid endarterectomy (CEA) functions better in the treating severe stenosis patients without clinical symptoms and moderate stenosis ones with symptoms. However, recent studies have suggested that carotid artery stenting (CAS) can also prevent high-risk carotid stenosis patients from stroke. In addition, CAS, which is advantageous in minor trauma and rapid recovery, has been applied more frequently.^3^ Therefore, the "gold standard" status of CEA is being threatened by CAS. This study aims to retrospectively analyze and to summarize the clinical effects and safety of CEA and CAS in the treatment of high-risk carotid stenosis patients as follows.

## METHODS

The patients who underwent CEA or CAS in our hospitals from January 2007 to December 2012 were selected in this study. Inclusion criteria: 1) 65-80 years old; 2) bilateral or unilateral stenosis; 3) carotid stenosis ≤70% without clinical symptoms; 4) carotid stenosis >50% with clinical symptoms.^4^ Exclusion criteria: 1) patients who were suffering from intracranial hemorrhage or whose intracranial hemorrhage occurred in recent three months, or those suffering from fresh lesions of cerebral infarction; 2) patients with blood pressures that were not maintained ideally; 3) patients who were prone to hemorrhage; 4) patients with complete occlusion of carotid artery; 5) stenosis was located in distal site, which was touchable by intervention or surgery; 6) patients with intracranial aneurysm which could not be treated simultaneously or in advance; 7) patients with surgical contraindications; (8) patients with malignant tumors and other malignant diseases. All patients were initially diagnosed as carotid stenosis by CTA or MRA, and after cerebral and aortic arch angiography, the disease was diagnosed as atherosclerotic common carotid artery bifurcation stenosis by neurologists and neurosurgeons. 74 cases of stenosis in 63 patients were enrolled. 35 patients were selected to undergo CEA as the control group, and 28 patients received CAS as the observation group. No significant differences were found in the general information such as age, gender composition, complications and degree of carotid stenosis between the two groups (P>0.05) (Table-I).

***Methods: ***Observation group (CAS): All patients were orally administered with 75 mg/d clopidogrel + 300 mg/d aspirin enteric-coated tablets before surgery for anticoagulant treatment. Local anesthesia was administered. A distal protection device was placed in the relatively straight site in carotid artery without lesions in its rock section, but notably, distal carotid artery lesion should be kept at least 3 cm away from the proximal protection device. Before stent implant, coronary balloon with appropriate size was commonly used to predilate the lesion area. According to the results of cerebral angiography, a stent diametered about 2 mm longer than that of carotid artery was implanted to cover the lesion completely. Continuous heparin sodium was given intraoperatively by intravenous injection. The patients were given 300 mg/d aspirin enteric-coated tablets + 75 mg/d clopidogrel within 3 months after surgery, and were treated only with 100 mg/d aspirin enteric-coated tablets + 75 mg/d clopidogrel 3 months later.

*Control group (CEA):* General anesthesia was performed after endotracheal intubation. The incision site should be located in the anterior border of sternocleidomastoid muscle. The compensation of collateral circulation should be continuously determined by Doppler ultrasound in the midst of surgery. Of the 35 patients undergoing CEA, 12 experienced the decrease of arterial blood velocity to 40% or below after internal carotid occlusion, for whom vascular shunt was adopted for treatment. To prevent the formation of postoperative arterial thrombosis, 4000 U of heparin sodium should be intravenously injected 5 min before internal carotid occlusion. After the external and internal carotid arteries and branch of common carotid artery were occluded temporarily, the patients were successively subjected to carotid artery incision, plaque removal and cleaning, suture and fixation. During surgery, five patients succumbed to small distal internal carotid artery, on whom carotid patch was used for appropriate repair and expansion. All patients were required to take lifelong 100 mg/d aspirin enteric-coated tablets for postoperative anticoagulant treatment.

***Observation Endpoints: ***Primary endpoints of observation: adverse events of cardiovascular diseases, death and stroke within 1 month of interventional or surgical treatment; ipsilateral stroke or death during the 6 months of follow-up.

Secondary endpoints of observation: interventional or surgical and perioperative complications such as hyperperfusion syndrome, hemodynamic disorders, local hematoma, peripheral nerve injury, or acute carotid occlusion.

***Follow-up: ***DSA, CTA and neck B ultrasonic examination were conducted one week after surgery to comprehensively determine the short-term clinical efficacy. Neurological examination, DSA, CTA and neck B ultrasound were performed one month, 6 and 12 months after surgery. The patients were rechecked and followed-up for three consecutive years.

***Statistical Analysis: ***Data were collected and analyzed by SPSS 17.0. The measurement data were expressed as (x±s), and t test was used for inter-group comparison. The numeration data were expressed as n (number of cases) and % (percentage), and χ^2^ test was used for inter-group comparison. P<0.05 was considered statistically significant.

## RESULTS

**Fig.1:**
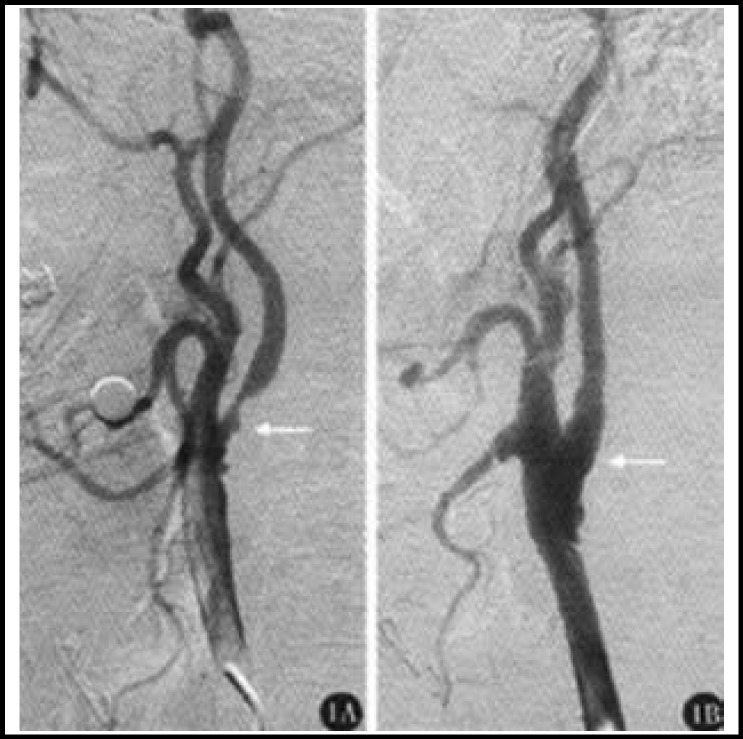
Control group. A: Severe stenosis of left internal carotid artery (threshold part) accompanied by ulcers before surgery; B: Stenosis of left internal carotid artery (threshold part) without ulcers after CEA

**Fig.2:**
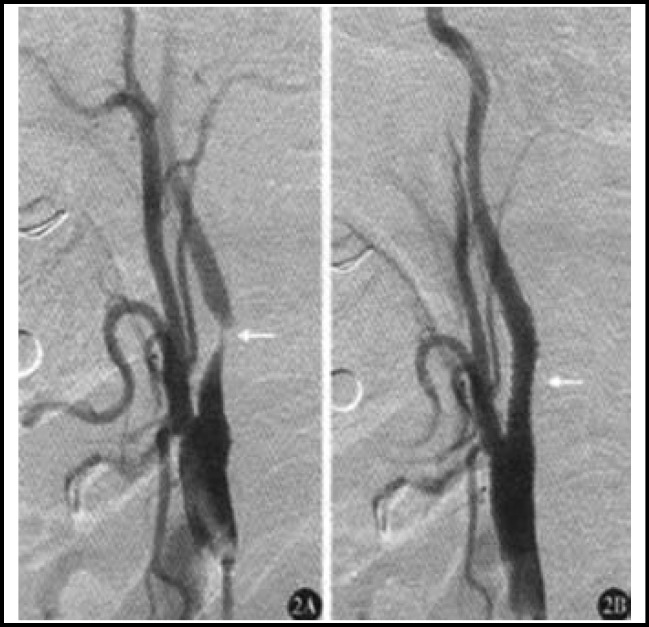
Observation group. A: Severe stenosis of right internal carotid artery before surgery; B: Vanished stenosis after CAS

***Comparison between the Two Groups: ***In the observation group, two patients suffered from transient cerebral ischemia during the perioperative period, which was mainly attributed to the placement of distal protection device. After the device was removed, the symptoms of cerebral ischemia were significantly alleviated. Three patients experienced postoperative hypotension, which was relieved after being treated with dopamine. The mean follow-up time of the 28 patients was (12.58±2.95) months, and no patient underwent recurrent carotid restenosis except for a case of cerebral ischemia. In the control group, a patient died of respiratory failure in the perioperative period. Acute carotid thrombosis appeared in one patient who succumbed to mild paralysis on the right upper limb after the embolization was removed by thrombolysis. The mean follow-up time of the 35 patients was (12.48±3.12) months, during which a patient suffering carotid stenosis again was engaged in treatment (Fig.1 and Fig.2).

**Table-I:** General information of the two groups

*Group*	*Case No.* *(n)*	*Age* *(Years old)*	*Gender*		*Complication*				*Degree of carotid stenosis*
			*Male*	*Female*	*Diabetes*	*Hypertension*	*Coronary artery disease*	*Hyperlipidemia*	
Observation	28	68.32±6.25	19	9	20	19	6	14	73.58±6.42
Control	35	69.96±7.01	23	12	24	23	7	17	73.39±5.11

**Table-II:** Primary endpoints of the two groups

*Group*	*Case No.* *(n)*	*Postoperative 1st month*			*Postoperative 6th month*		*Total*
		*Adverse cardiac events*	*Stroke*	*Death*	*Ipsilateral stroke event*	*Death*	
Observation	28	0	2	0	1	0	2 (7.14)
Control	35	0	1	1	1	0	4 (11.43)

**Table-III:** Secondary endpoints of the two groups

*Group*	*Case No. (n)*	*Recurrent severe stenosis in postoperative 1st year*	*Hemodynamic disorder*	*Hyperperfusion syndrome*	*Local hematoma*	*Peripheral nerve injury*	*Acute carotid occlusion*	*Total*
Observation	28	0	3	0	0	0	0	3 (10.71)
Control	35	1	1	0	0	1	1	4 (11.43)

***Primary and Secondary Endpoints of the Two Groups: ***Two (7.14%) and three (10.71%) patients in the observation group reached primary and secondary endpoints respectively, while 4 patients each (11.43%) in the control group reached primary and secondary endpoints respectively. The arrival rates of primary and secondary endpoints of the two groups did not differ significantly (P>0.05) (Table-II and Table-III).

## DISCUSSION

Carotid artery diseases are ubiquitous in contemporary society, which inevitably bring about cardiovascular and cerebrovascular diseases. Authoritative institutions indicate that carotid stenosis (>70%) can be diagnosed if the peak systolic velocity of internal carotid exceeds 230 cm/s and intravascular atherosclerotic plaques can be observed by ultrasound examination. Another study shows that carotid stenosis can be confirmed in case the peak systolic velocity is higher than 230 cm/s, the end-diastolic velocity exceeds 100 cm/s, or the ratios of peak systolic velocity to normal one is higher than three.

Randomized controlled clinical research trials have shown that^5^ CEA and other revascularization protocols can benefit patients. Meta-analysis suggests that when the degree of carotid stenosis surpasses 50%, CEA treatment can significantly decrease postoperative mortality rate and the incidence of stroke in patients. Compared with the patients with 50%-69% of stenosis, those with 70%-99% of stenosis may gain more benefits. The perioperative mortality and (or) incidence of stroke basically resembled at about 6% in all literatures analyzed. When the carotid stenosis of patients exceeds 60%, CEA can also significantly lower its mortality rate and incidence of stroke than oral medication.^6^^-^^8^ In this study, a patient directly died of respiratory failure during perioperative period (2.86%) owing to the complicated pulmonary fibrosis. Therefore, the death was not obviously associated with CEA, which, however, reminds us paying attention to the preoperative evaluation on high-risk factors, aiming to ensure the safety of patients and smooth surgery. The incidence rate of CEA complications was 11.43%, and no stroke event attacked most patients during follow-up, indicating the curative effects of CEA on the treatment of high-risk carotid stenosis patients. In this study, both the efficacy and safety of CEA are consistent with the results of several randomized controlled clinical trials.^9^^-^^11^

CAS has been applied in clinic since the 1990s. The clinical use of distal embolic protection devices and special tools for self-expanding stent has enabled CAS to be increasingly applied in treating high-risk carotid stenosis. CAS carrying self-expanding stents and distal embolic protection devices has become one of the representative surgical means of carotid revascularization.^12^^-^^14^ In this study, all patients were successfully implanted with stents without complications such as hyperperfusion syndrome, local hematoma, peripheral nerve injury, and acute occlusion of carotid artery, etc. Two cases of patients appeared transient symptoms of cerebral ischemia.^15^^,^^16^ The results of this study suggest that CAS can definitely treat high-risk carotid stenosis effectively and securely.

The safety of CAS and CEA in treating high-risk carotid stenosis was compared by observing primary endpoints, such as the adverse events of cardiovascular diseases, stroke and death, which did not differ significantly in the two groups. The efficacies of the two treatment protocols were also compared by observing secondary endpoints such as 1-year postoperative severe restenosis, hemodynamic disorders and high-perfusion syndrome, which did not differ significantly in the two groups either.

In summary, the efficacies and safety of CAS and CEA are similar in the treatment of high-risk carotid stenosis patients, but the retrospective analysis herein is merely based on a small sample size. Therefore, prospective and multi-center randomized controlled trials with large sample sizes are still needed for further issues.

## Authors Contributions:

**WPF:** Designed the protocol.

**LCY and DJC: **Clinical data collection and experiments.

**LJL:** Prepared the final manuscript.
